# Supplementary material for: Deep generative computed perfusion-deficit mapping of ischaemic stroke
Source: Commun Biol. 2026 Feb 4;9:219. doi: 10.1038/s42003-025-09495-6 (PMC12894690; doi:10.1038/s42003-025-09495-6)
Supplement: Supplementary file 2 — Supplementary Informations [file 42003_2025_9495_MOESM2_ESM.pdf]

Supplementary Figures

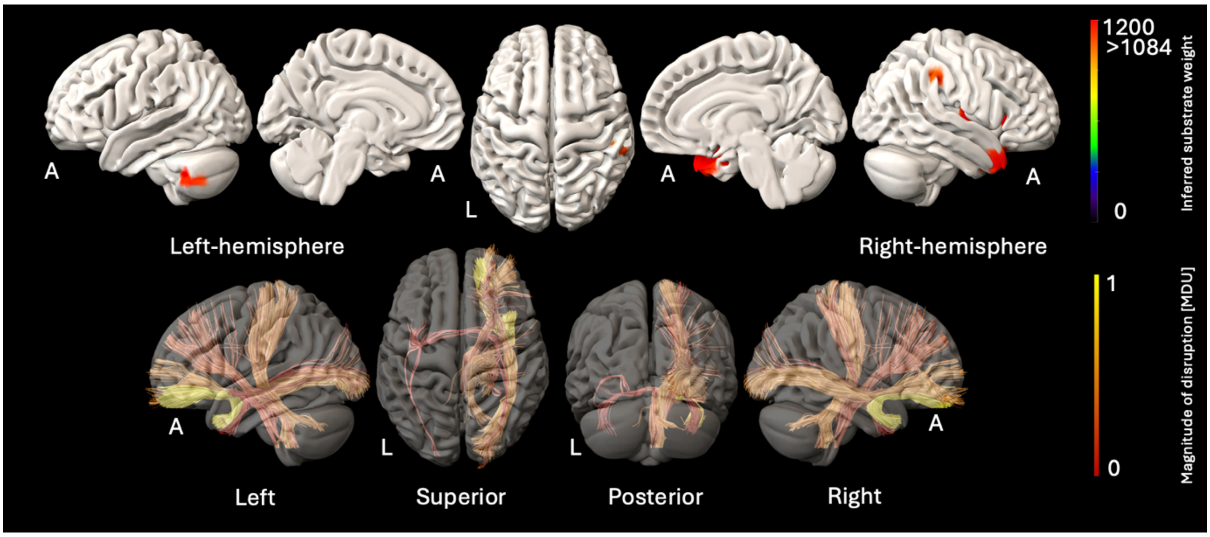

Supplementary Figure 1: Grey and white matter substrates underlying left hand deficit. (Row 1) GM perfusion-deficit maps for left hand. The colour bar indicates the weight of association between each brain area and the NIHSS left hand score. Voxels with weights exceeding 1084 are considered significant. (Row 2) Disrupted WM tracts for left hand, with the colour bar indicating normalized magnitude of disruption, scaled from 0 to 1 [MDU: Magnitude disruption unit].

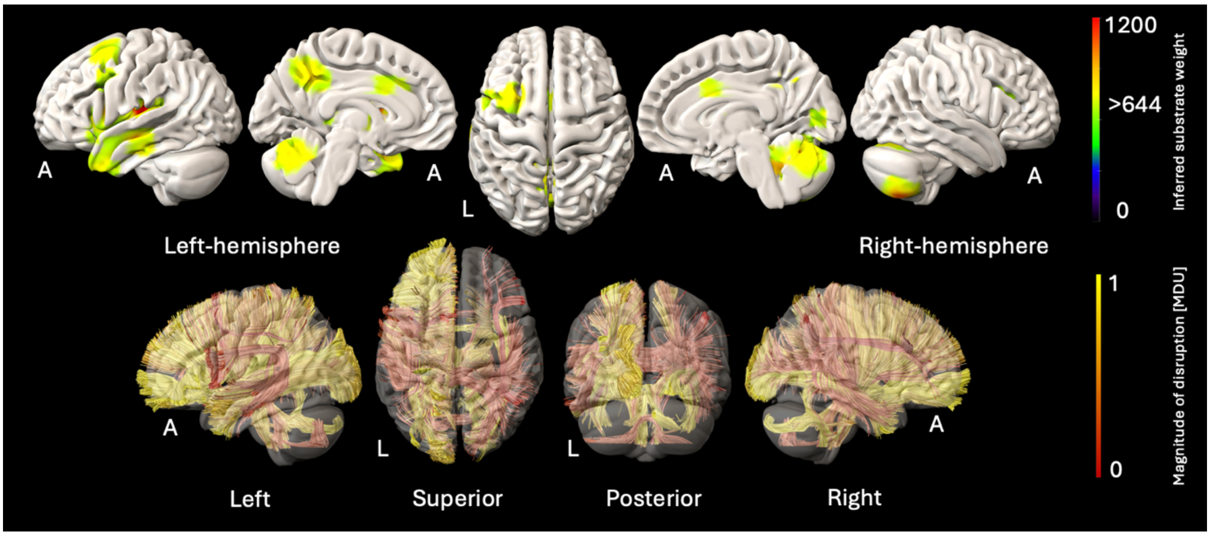

Supplementary Figure 2: Grey and white matter substrates underlying right hand deficit. (Row 1) GM perfusion-deficit maps for right hand. The colour bar indicates the weight of association between each brain area and the NIHSS right hand score. Voxels with weights exceeding 644 are considered significant. (Row 2) Disrupted WM tracts for right hand, with the colour bar indicating normalized magnitude of disruption, scaled from 0 to 1 [MDU: Magnitude disruption unit].

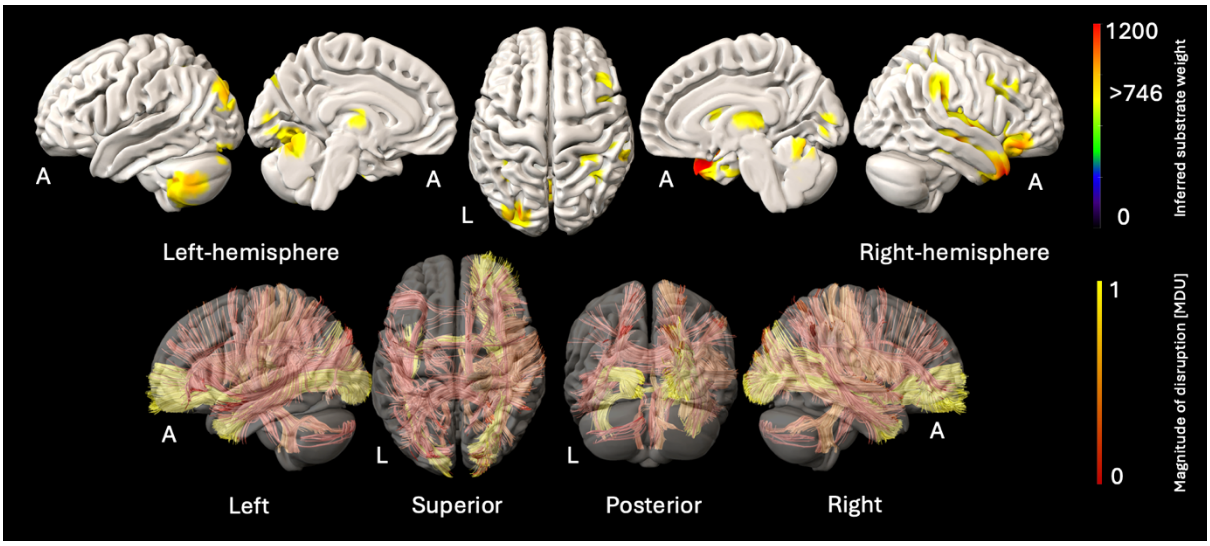

Supplementary Figure 3: Grey and white matter substrates underlying left leg deficit. (Row 1) GM perfusion-deficit maps for left leg. The colour bar indicates the weight of association between each brain area and the NIHSS left leg score. Voxels with weights exceeding 746 are considered significant. (Row 2) Disrupted WM tracts for left leg, with the colour bar indicating normalized magnitude of disruption, scaled from 0 to 1 [MDU: Magnitude disruption unit].

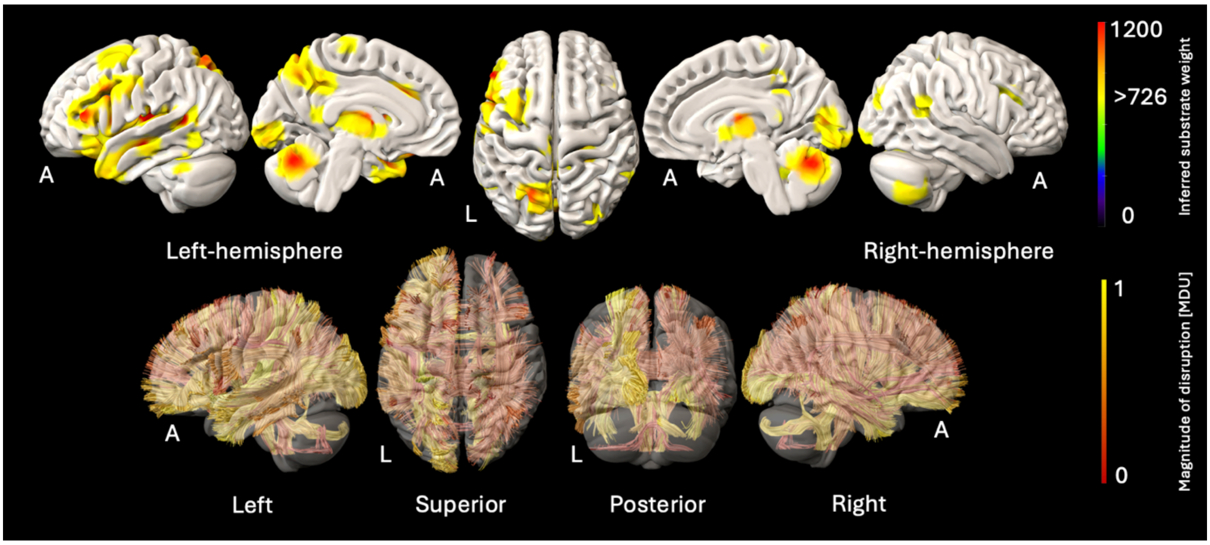

Supplementary Figure 4: Grey and white matter substrates underlying right leg deficit. (Row 1) GM perfusion-deficit maps for right leg. The colour bar indicates the weight of association between each brain area and the NIHSS right leg score. Voxels with weights exceeding 726 are considered significant. (Row 2) Disrupted WM tracts for right leg, with the colour bar indicating normalized magnitude of disruption, scaled from 0 to 1 [MDU: Magnitude disruption unit].

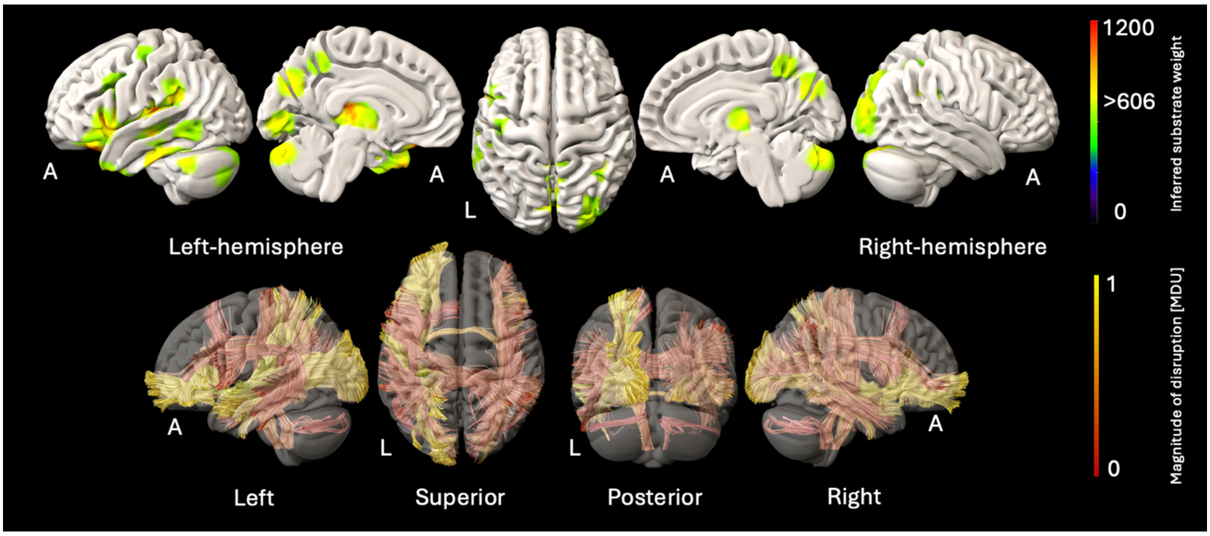

Supplementary Figure 5: Grey and white matter substrates underlying Loc-Question. (Row 1) GM perfusion-deficit maps for Loc-Question. The colour bar indicates the weight of association between each brain area and the NIHSS Loc-Question score. Voxels with weights exceeding 606 are considered significant. (Row 2) Disrupted WM tracts for Loc-Question, with the colour bar indicating normalized magnitude of disruption, scaled from 0 to 1 [MDU: Magnitude disruption unit].

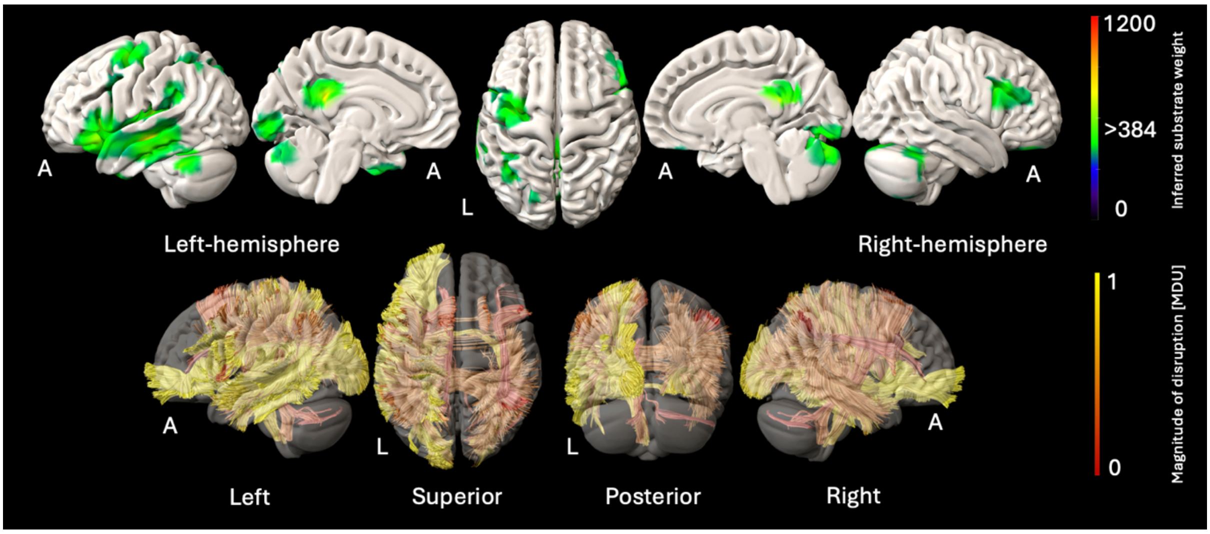

Supplementary Figure 6: Grey and white matter substrates underlying Loc-Command. (Row 1) GM perfusion-deficit maps for Loc-Command. The colour bar indicates the weight of association between each brain area and the NIHSS Loc-Command score. Voxels with weights exceeding 384 are considered significant. (Row 2) Disrupted WM tracts for Loc-Command, with the colour bar indicating normalized magnitude of disruption, scaled from 0 to 1 [MDU: Magnitude disruption unit].

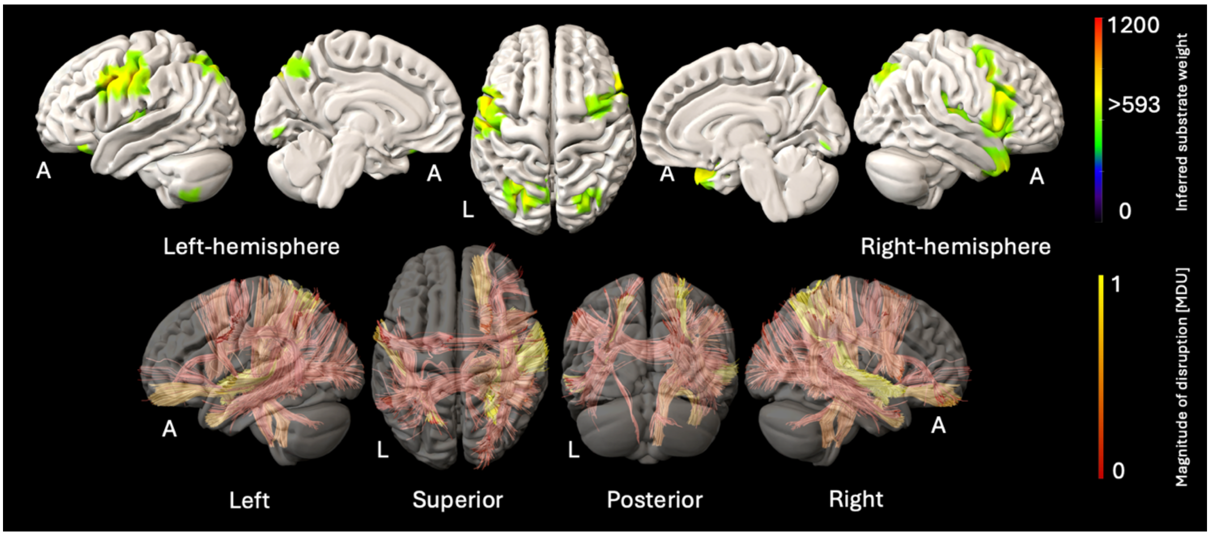

Supplementary Figure 7: Grey and white matter substrates underlying gaze. (Row 1) GM perfusion-deficit maps for gaze. The colour bar indicates the weight of association between each brain area and the NIHSS gaze score. Voxels with weights exceeding 593 are considered significant. (Row 2) Disrupted WM tracts for gaze, with the colour bar indicating normalized magnitude of disruption, scaled from 0 to 1 [MDU: Magnitude disruption unit].

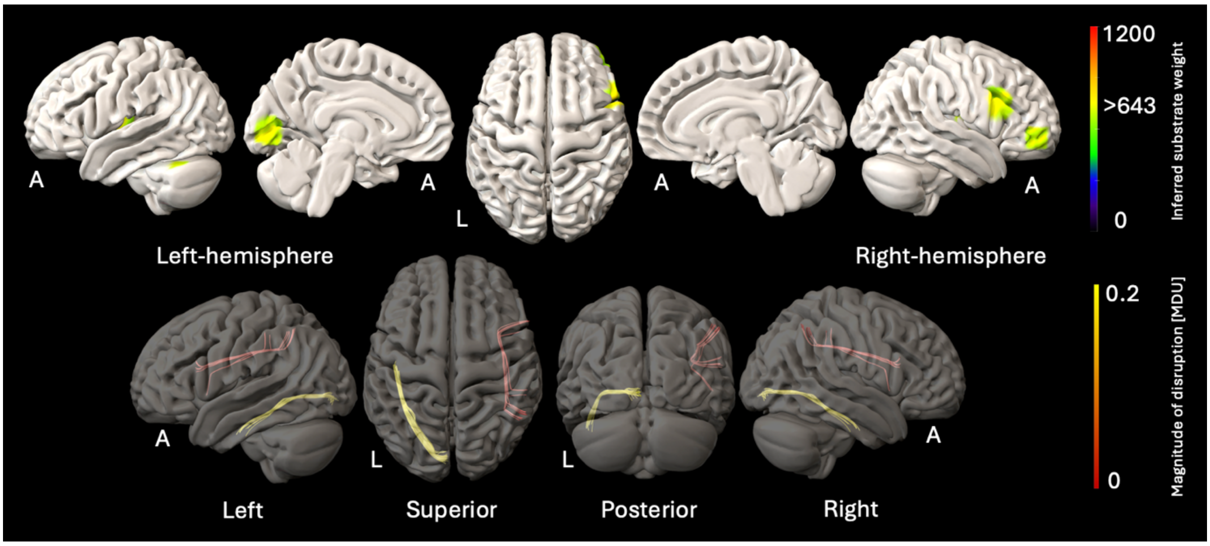

Supplementary Figure 8: Grey and white matter substrates underlying visual deficit. (Row 1) GM perfusion-deficit maps for visual. The colour bar indicates the weight of association between each brain area and the NIHSS visual score. Voxels with weights exceeding 643 are considered significant. (Row 2) Disrupted WM tracts for visual, with the colour bar indicating normalized magnitude of disruption, scaled from 0 to 0.2 [MDU: Magnitude disruption unit].

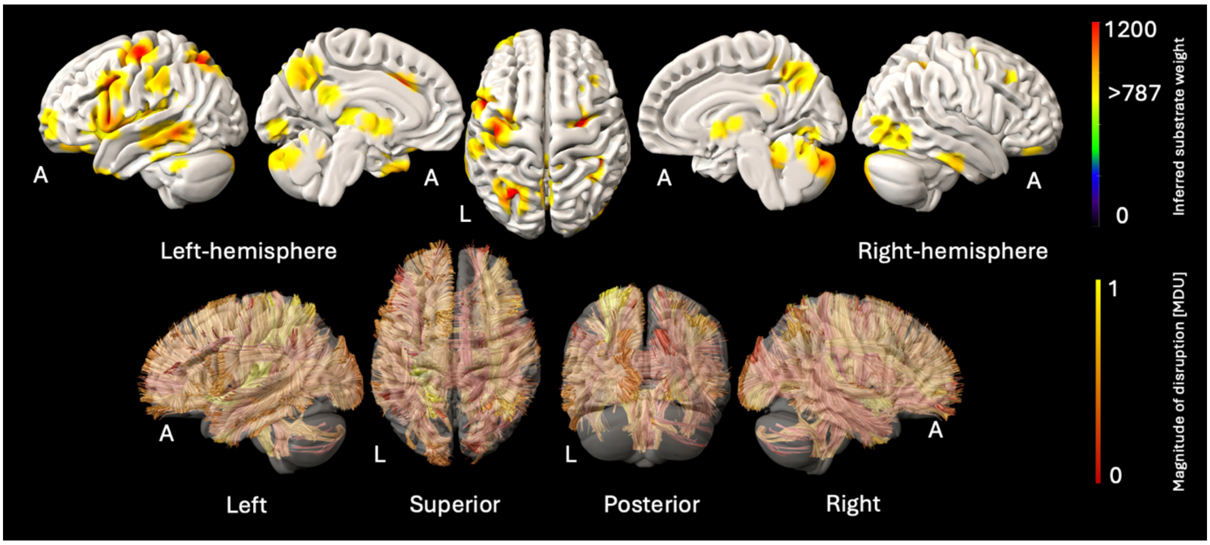

Supplementary Figure 9: Grey and white matter substrates underlying language deficit. (Row 1) GM perfusion-deficit maps for language. The colour bar indicates the weight of association between each brain area and the NIHSS language score. Voxels with weights exceeding 787 are considered significant. (Row 2) Disrupted WM tracts for language, with the colour bar indicating normalized magnitude of disruption, scaled from 0 to 1 [MDU: Magnitude disruption unit].

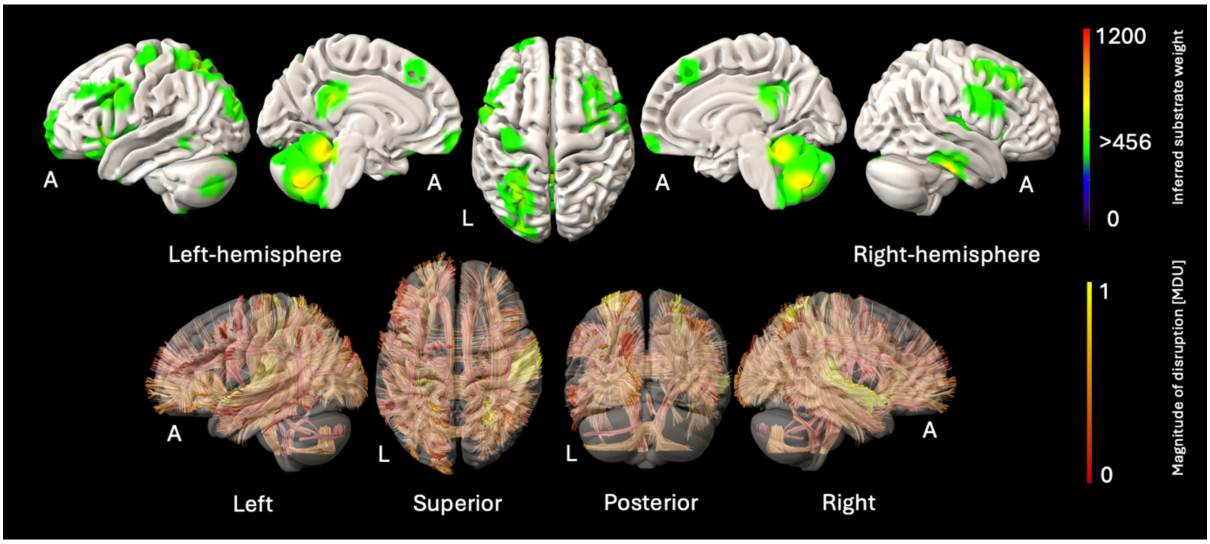

Supplementary Figure 10: Grey and white matter substrates underlying dysarthria. (Row 1) GM perfusion-deficit maps for dysarthria. The colour bar indicates the weight of association between each brain area and the NIHSS dysarthria score. Voxels with weights exceeding 456 are considered significant. (Row 2) Disrupted WM tracts for dysarthria, with the colour bar indicating normalized magnitude of disruption, scaled from 0 to 1 [MDU: Magnitude disruption unit].

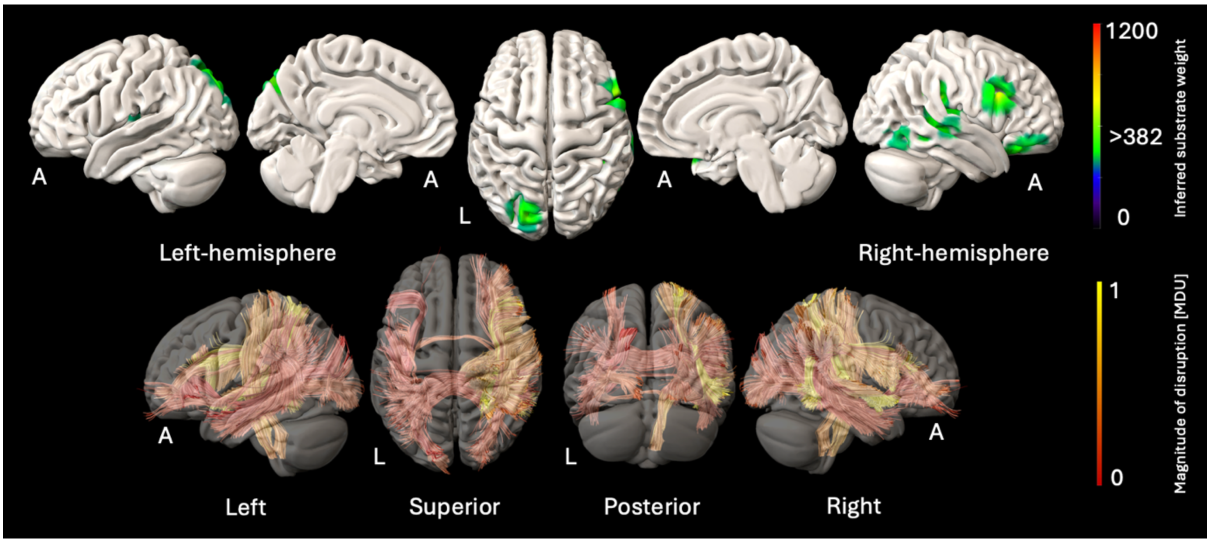

Supplementary Figure 11: Grey and white matter substrates underlying somatosensory deficit. (Row 1) GM perfusion-deficit maps for somatosensory. The colour bar indicates the weight of association between each brain area and the NIHSS somatosensory score. Voxels with weights exceeding 382 are considered significant. (Row 2) Disrupted WM tracts for somatosensory, with the colour bar indicating normalized magnitude of disruption, scaled from 0 to 1 [MDU: Magnitude disruption unit].

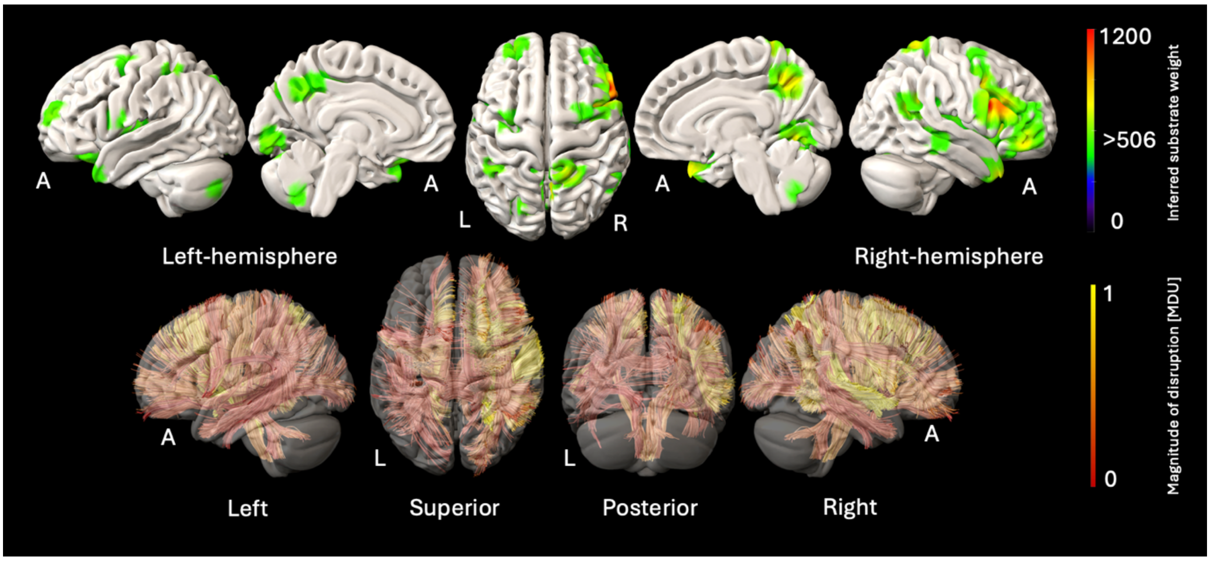

Supplementary Figure 12: Grey and white matter substrates underlying attention deficit. (Row 1) GM perfusion-deficit maps for attention. The colour bar indicates the weight of association between each brain area and the NIHSS attention score. Voxels with weights exceeding 506 are considered significant. (Row 2) Disrupted WM tracts for attention, with the colour bar indicating normalized magnitude of disruption, scaled from 0 to 1 [MDU: Magnitude disruption unit].

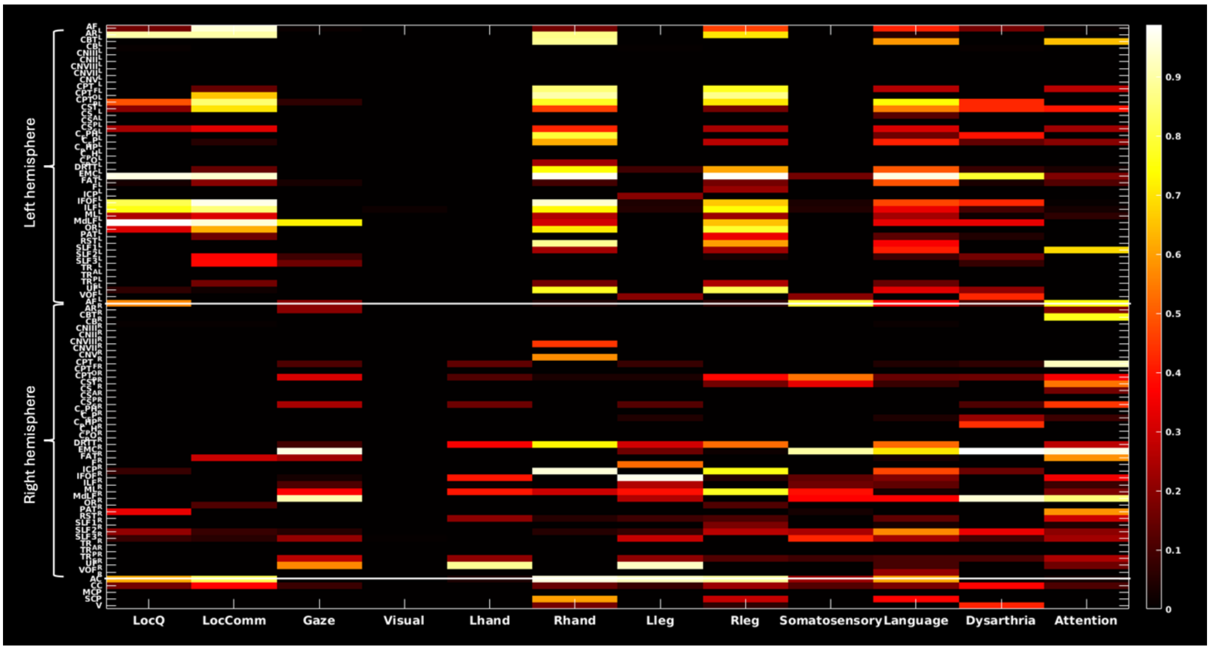

Supplementary Figure 13: Affected WM tract density across NIHSS sub-scores. Affected WM tract density is shown for each WM bundle across twelve NIHSS sub-scores (except for three negative substrates: Loc-Arrival, facial palsy, and ataxia). Note that: in the visual WM substrate, the disruptive density of all tracts is zero except for the left inferior longitudinal fasciculus (0.0174) and the right superior longitudinal fasciculus III (0.0065), respectively.

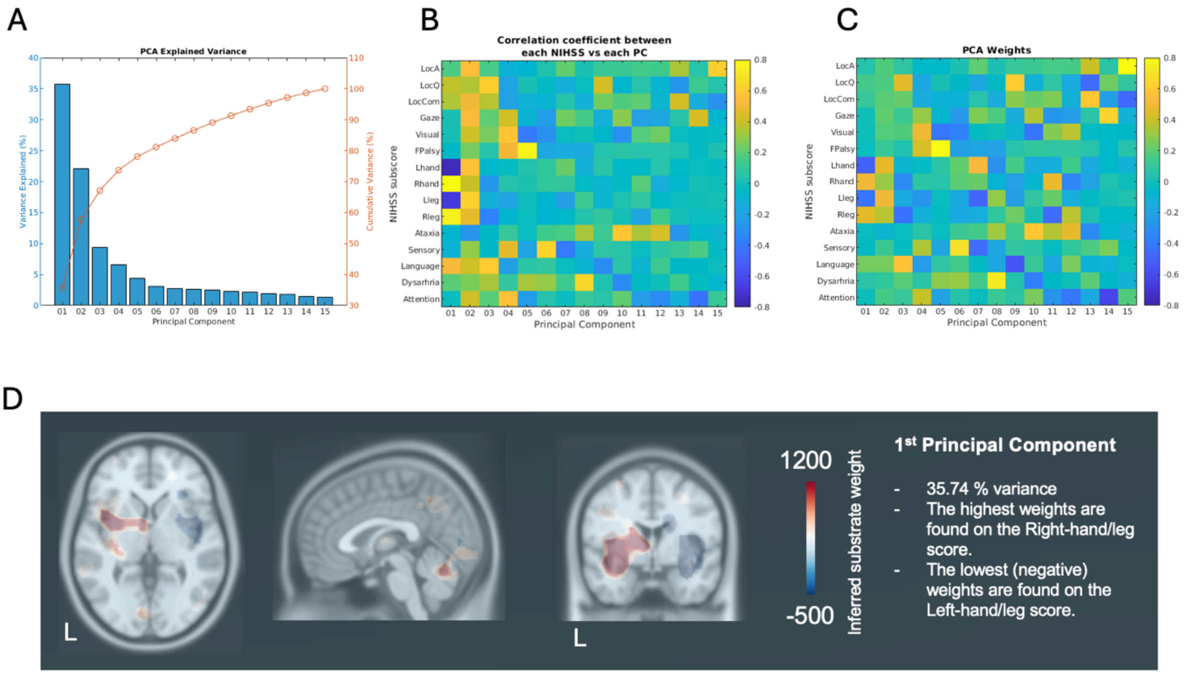

Supplementary Figure 14: Principal component analysis of NIHSS sub-scores. (A) Principal component analysis (PCA) of NIHSS sub-scores. (B) Correlation coefficients between each NIHSS sub-score and each principal component. (C) PCA weights for each NIHSS sub-score. (D) Superposition of all inferred substrate maps weighted by PCA loadings from the first principal component. This PCA-weighted superposition mask was generated as a linear combination of all NIHSS sub-score masks, weighted by their corresponding PCA loadings.

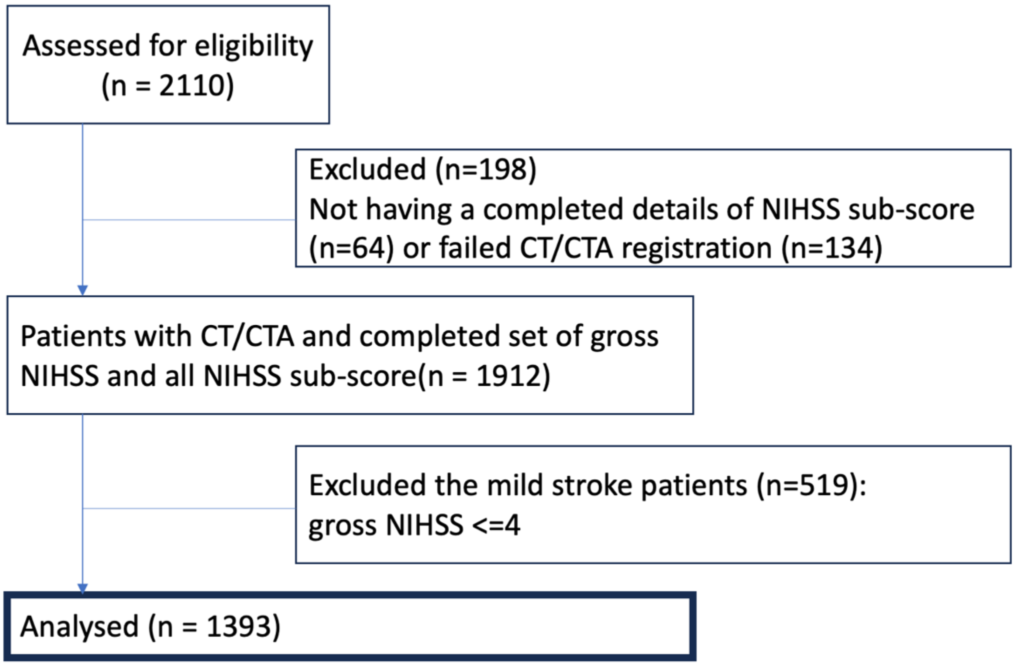

Supplementary Figure 15: Participant flowchart for the study.

Supplementary Tables

Supplementary Table 1: Disrupted white matter tracts of the left-hand WM mask.

Abbreviation	Full Name	Magnitude of disruption
AC	Anterior Commissure	0.04
CPT_F_R	Corticopontine Tract Frontal Right	0.14
CPT_P_R	Corticopontine Tract Parietal Right	0.11
CS_S_R	Corona Radiata Superior Right	0.16
DRTT_R	Dentatorubrothalamic Tract Right	0.36
IFOF_R	Inferior Fronto-occipital Fasciculus Right	0.40
ILF_R	Inferior Longitudinal Fasciculus Right	0.01
ML_R	Medial Lemniscus Right	0.40
RST_R	Rubrospinal Tract Right	0.20
TR_S_R	Thalamic Radiation Superior Right	0.21
UF_R	Uncinate Fasciculus Right	0.88

Supplementary Table 2: Disrupted white matter tracts of the right-hand WM mask.

Abbreviation	Full Name	Magnitude of disruption
AC	Anterior Commissure	0.95
AF_L	Arcuate Fasciculus Left	0.17
AF_R	Arcuate Fasciculus Right	0.04
AR_L	Acoustic Radiation Left	0.87
CBT_L	Corticobulbar Tract Left	0.88
CC	Corpus Callosum	0.15
CNVIII_R	Vestibulocochlear Nerve Right	0.44
CNV_R	Trigeminal Nerve Right	0.57
CPT_F_L	Corticopontine Tract Frontal Left	0.86
CPT_O_L	Corticopontine Tract Occipital Left	0.91
CPT_P_L	Corticopontine Tract Parietal Left	0.78
CPT_P_R	Corticopontine Tract Parietal Right	0.04
CST_L	Corticospinal Tract Left	0.46
CS_S_L	Corona Radiata Superior Left	0.42
C_FPH_L	Cingulum Frontal Parahippocampal Left	0.79
C_FP_L	Cingulum Frontal Parietal Left	0.62
C_PO_L	Cingulum Parietal Occipital Left	0.23
DRTT_L	Dentatorubrothalamic Tract Left	0.74
DRTT_R	Dentatorubrothalamic Tract Right	0.73
EMC_L	Extreme Capsule Left	0.97
FAT_L	Frontal Aslant Tract Left	0.09
ICP_R	Inferior Cerebellar Peduncle Right	0.95
IFOF_L	Inferior Fronto-occipital Fasciculus Left	0.94
ILF_L	Inferior Longitudinal Fasciculus Left	0.73
ML_L	Medial Lemniscus Left	0.26
ML_R	Medial Lemniscus Right	0.29
MdLF_L	Middle Longitudinal Fasciculus Left	0.30
OR_L	Optic Radiation Left	0.71
RST_L	Rubrospinal Tract Left	0.88
RST_R	Rubrospinal Tract Right	0.05
SCP	Superior Cerebellar Peduncle	0.60
SLF1_L	Superior Longitudinal Fasciculus I Left	0.24
SLF2_L	Superior Longitudinal Fasciculus II Left	0.01
SLF2_R	Superior Longitudinal Fasciculus II Right	0.05
TR_S_L	Thalamic Radiation Superior Left	0.17
UF_L	Uncinate Fasciculus Left	0.77
V	Vermis	0.17

Supplementary Table 3: Disrupted white matter tracts of the left-leg WM mask.

Abbreviation	Full Name	Magnitude of disruption
AC	Anterior Commissure	0.92
CB_L	Cingulum Bundle Left	0.01
CC	Corpus Callosum	0.08
CPT_F_R	Corticopontine Tract Frontal Right	0.08
CPT_P_R	Corticopontine Tract Parietal Right	0.04
CS_S_R	Corona Radiata Superior Right	0.12
C_FP_R	Cingulum Frontal Parietal Right	0.04
DRTT_L	Dentatorubrothalamic Tract Left	0.09
DRTT_R	Dentatorubrothalamic Tract Right	0.30
EMC_R	Extreme Capsule Right	0.16
F_R	Fornix Right	0.52
ICP_L	Inferior Cerebellar Peduncle Left	0.19
IFOF_L	Inferior Fronto-occipital Fasciculus Left	0.04
IFOF_R	Inferior Fronto-occipital Fasciculus Right	0.97
ILF_L	Inferior Longitudinal Fasciculus Left	0.05
ILF_R	Inferior Longitudinal Fasciculus Right	0.26
ML_R	Medial Lemniscus Right	0.39
MdLF_R	Middle Longitudinal Fasciculus Right	0.26
RST_R	Rubrospinal Tract Right	0.09
SLF2_R	Superior Longitudinal Fasciculus II Right	0.07
SLF3_R	Superior Longitudinal Fasciculus III Right	0.29
TR_S_R	Thalamic Radiation Superior Right	0.21
UF_R	Uncinate Fasciculus Right	0.93
VOF_L	Vertical Occipital Fasciculus Left	0.19

Supplementary Table 4: Disrupted white matter tracts of the right-leg WM mask.

Abbreviation	Full Name	Magnitude of disruption
AC	Anterior Commissure	0.89
AF_L	Arcuate Fasciculus Left	0.46
AF_R	Arcuate Fasciculus Right	0.06
AR_L	Acoustic Radiation Left	0.70
CC	Corpus Callosum	0.22
CPT_F_L	Corticopontine Tract Frontal Left	0.77
CPT_O_L	Corticopontine Tract Occipital Left	0.88
CPT_P_L	Corticopontine Tract Parietal Left	0.71
CPT_P_R	Corticopontine Tract Parietal Right	0.37
CST_L	Corticospinal Tract Left	0.16
CST_R	Corticospinal Tract Right	0.16
CS_S_L	Corona Radiata Superior Left	0.24
C_FP_L	Cingulum Frontal Parietal Left	0.27
DRTT_L	Dentatorubrothalamic Tract Left	0.61
DRTT_R	Dentatorubrothalamic Tract Right	0.53
EMC_L	Extreme Capsule Left	0.98
EMC_R	Extreme Capsule Right	0.02
FAT_L	Frontal Aslant Tract Left	0.17
F_L	Fornix Left	0.22
ICP_R	Inferior Cerebellar Peduncle Right	0.76
IFOF_L	Inferior Fronto-occipital Fasciculus Left	0.66
IFOF_R	Inferior Fronto-occipital Fasciculus Right	0.05
ILF_L	Inferior Longitudinal Fasciculus Left	0.75
ML_L	Medial Lemniscus Left	0.23
ML_R	Medial Lemniscus Right	0.77
MdLF_L	Middle Longitudinal Fasciculus Left	0.65
OR_L	Optic Radiation Left	0.79
OR_R	Optic Radiation Right	0.11
PAT_L	Parietoangular Tract Left	0.34
RST_L	Rubrospinal Tract Left	0.60
RST_R	Rubrospinal Tract Right	0.11
SCP	Superior Cerebellar Peduncle	0.29
SLF1_L	Superior Longitudinal Fasciculus I Left	0.23
SLF1_R	Superior Longitudinal Fasciculus I Right	0.18
SLF2_L	Superior Longitudinal Fasciculus II Left	0.03
SLF2_R	Superior Longitudinal Fasciculus II Right	0.27
TR_S_L	Thalamic Radiation Superior Left	0.25
TR_S_R	Thalamic Radiation Superior Right	0.11
UF_L	Uncinate Fasciculus Left	0.82
V	Vermis	0.06

Supplementary Table 5: Disrupted white matter tracts of the Loc-Question WM mask.

Abbreviation	Full Name	Magnitude of disruption
AC	Anterior Commissure	0.62
AF_L	Arcuate Fasciculus Left	0.16
AF_R	Arcuate Fasciculus Right	0.57
AR_L	Acoustic Radiation Left	0.90
CB_L	Cingulum Bundle Left	0.01
CB_R	Cingulum Bundle Right	0.01
CC	Corpus Callosum	0.16
CPT_P_L	Corticopontine Tract Parietal Left	0.49
CST_L	Corticospinal Tract Left	0.20
CS_S_L	Corona Radiata Superior Left	0.24
EMC_L	Extreme Capsule Left	0.96
FAT_L	Frontal Aslant Tract Left	0.03
ICP_R	Inferior Cerebellar Peduncle Right	0.08
IFOF_L	Inferior Fronto-occipital Fasciculus Left	0.82
ILF_L	Inferior Longitudinal Fasciculus Left	0.76
ML_L	Medial Lemniscus Left	0.25
MdLF_L	Middle Longitudinal Fasciculus Left	0.99
OR_L	Optic Radiation Left	0.32
PAT_R	Parietoangular Tract Right	0.34
SLF2_R	Superior Longitudinal Fasciculus II Right	0.21
SLF3_R	Superior Longitudinal Fasciculus III Right	0.08
UF_L	Uncinate Fasciculus Left	0.07

Supplementary Table 6: Disrupted white matter tracts of the Loc-Command WM mask.

Abbreviation	Full Name	Magnitude of disruption
AC	Anterior Commissure	0.82
AF_L	Arcuate Fasciculus Left	0.95
AR_L	Acoustic Radiation Left	0.90
CB_R	Cingulum Bundle Right	0.01
CC	Corpus Callosum	0.37
CPT_F_L	Corticopontine Tract Frontal Left	0.14
CPT_O_L	Corticopontine Tract Occipital Left	0.66
CPT_P_L	Corticopontine Tract Parietal Left	0.85
CST_L	Corticospinal Tract Left	0.70
CS_S_L	Corona Radiata Superior Left	0.33
C_FP_L	Cingulum Frontal Parietal Left	0.05
DRTT_L	Dentatorubrothalamic Tract Left	0.14
EMC_L	Extreme Capsule Left	0.94
FAT_L	Frontal Aslant Tract Left	0.20
FAT_R	Frontal Aslant Tract Right	0.29
IFOF_L	Inferior Fronto-occipital Fasciculus Left	0.96
ILF_L	Inferior Longitudinal Fasciculus Left	0.85
ML_L	Medial Lemniscus Left	0.32
MdLF_L	Middle Longitudinal Fasciculus Left	0.95
OR_L	Optic Radiation Left	0.62
OR_R	Optic Radiation Right	0.11
PAT_L	Parietoangular Tract Left	0.16
SLF2_L	Superior Longitudinal Fasciculus II Left	0.37
SLF2_R	Superior Longitudinal Fasciculus II Right	0.08
SLF3_L	Superior Longitudinal Fasciculus III Left	0.38
SLF3_R	Superior Longitudinal Fasciculus III Right	0.05
TR_S_L	Thalamic Radiation Superior Left	0.17
UF_L	Uncinate Fasciculus Left	0.02

Supplementary Table 7: Disrupted white matter tracts of the gaze WM mask.

Abbreviation	Full Name	Magnitude of disruption
AF_L	Arcuate Fasciculus Left	0.01
AF_R	Arcuate Fasciculus Right	0.20
AR_R	Acoustic Radiation Right	0.20
CC	Corpus Callosum	0.08
CPT_F_R	Corticopontine Tract Frontal Right	0.11
CPT_P_L	Corticopontine Tract Parietal Left	0.07
CPT_P_R	Corticopontine Tract Parietal Right	0.31
CS_S_R	Corona Radiata Superior Right	0.24
DRTT_R	Dentatorubrothalamic Tract Right	0.09
EMC_R	Extreme Capsule Right	0.96
FAT_L	Frontal Aslant Tract Left	0.03
FAT_R	Frontal Aslant Tract Right	0.23
IFOF_R	Inferior Fronto-occipital Fasciculus Right	0.03
ILF_R	Inferior Longitudinal Fasciculus Right	0.11
ML_R	Medial Lemniscus Right	0.36
MdLF_L	Middle Longitudinal Fasciculus Left	0.72
MdLF_R	Middle Longitudinal Fasciculus Right	0.91
SLF2_L	Superior Longitudinal Fasciculus II Left	0.09
SLF2_R	Superior Longitudinal Fasciculus II Right	0.05
SLF3_L	Superior Longitudinal Fasciculus III Left	0.16
SLF3_R	Superior Longitudinal Fasciculus III Right	0.22
TR_S_R	Thalamic Radiation Superior Right	0.27
UF_R	Uncinate Fasciculus Right	0.57

Supplementary Table 8: Disrupted white matter tracts of the visual WM mask.

Abbreviation	Full Name	Magnitude of disruption
ILF_L	Inferior Longitudinal Fasciculus Left	0.02
SLF3_R	Superior Longitudinal Fasciculus III Right	0.01

Supplementary Table 9: Disrupted white matter tracts of the language WM mask.

Abbreviation	Full Name	Magnitude of disruption
AC	Anterior Commissure	0.61
AF_L	Arcuate Fasciculus Left	0.42
AF_R	Arcuate Fasciculus Right	0.36
AR_L	Acoustic Radiation Left	0.05
CBT_L	Corticobulbar Tract Left	0.59
CB_R	Cingulum Bundle Right	0.01
CC	Corpus Callosum	0.18
CPT_F_L	Corticopontine Tract Frontal Left	0.26
CPT_F_R	Corticopontine Tract Frontal Right	0.05
CPT_P_L	Corticopontine Tract Parietal Left	0.75
CPT_P_R	Corticopontine Tract Parietal Right	0.15
CST_L	Corticospinal Tract Left	0.56
CST_R	Corticospinal Tract Right	0.08
CS_A_L	Corona Radiata Anterior Left	0.13
CS_S_L	Corona Radiata Superior Left	0.32
C_FPH_L	Cingulum Frontal Parahippocampal Left	0.16
C_FP_L	Cingulum Frontal Parietal Left	0.41
C_FP_R	Cingulum Frontal Parietal Right	0.04
DRTT_L	Dentatorubrothalamic Tract Left	0.49
DRTT_R	Dentatorubrothalamic Tract Right	0.52
EMC_L	Extreme Capsule Left	0.96
EMC_R	Extreme Capsule Right	0.71
FAT_L	Frontal Aslant Tract Left	0.49
FAT_R	Frontal Aslant Tract Right	0.03
ICP_R	Inferior Cerebellar Peduncle Right	0.47
IFOF_L	Inferior Fronto-occipital Fasciculus Left	0.47
IFOF_R	Inferior Fronto-occipital Fasciculus Right	0.18
ILF_L	Inferior Longitudinal Fasciculus Left	0.34
ILF_R	Inferior Longitudinal Fasciculus Right	0.14
ML_L	Medial Lemniscus Left	0.24
ML_R	Medial Lemniscus Right	0.03
MdLF_L	Middle Longitudinal Fasciculus Left	0.34
MdLF_R	Middle Longitudinal Fasciculus Right	0.37
PAT_L	Parietoangular Tract Left	0.13
RST_L	Rubrospinal Tract Left	0.36
RST_R	Rubrospinal Tract Right	0.14
SCP	Superior Cerebellar Peduncle	0.37
SLF1_L	Superior Longitudinal Fasciculus I Left	0.41
SLF1_R	Superior Longitudinal Fasciculus I Right	0.03
SLF2_L	Superior Longitudinal Fasciculus II Left	0.09
SLF2_R	Superior Longitudinal Fasciculus II Right	0.56
SLF3_R	Superior Longitudinal Fasciculus III Right	0.23
TR_S_L	Thalamic Radiation Superior Left	0.15
TR_S_R	Thalamic Radiation Superior Right	0.10
UF_L	Uncinate Fasciculus Left	0.33
UF_R	Uncinate Fasciculus Right	0.10
VOF_R	Vertical Occipital Fasciculus Right	0.21

Supplementary Table 10: Disrupted white matter tracts of the dysarthria WM mask.

Abbreviation	Full Name	Magnitude of disruption
AF_L	Arcuate Fasciculus Left	0.17
AF_R	Arcuate Fasciculus Right	0.12
CB_L	Cingulum Bundle Left	0.01
CC	Corpus Callosum	0.37
CPT_F_R	Corticopontine Tract Frontal Right	0.06
CPT_P_L	Corticopontine Tract Parietal Left	0.43
CPT_P_R	Corticopontine Tract Parietal Right	0.16
CST_L	Corticospinal Tract Left	0.42
CS_S_R	Corona Radiata Superior Right	0.11
C_FPH_L	Cingulum Frontal Parahippocampal Left	0.40
C_FP_L	Cingulum Frontal Parietal Left	0.14
C_FP_R	Cingulum Frontal Parietal Right	0.22
C_PHP_R	Cingulum Parahippocampal Parietal Right	0.44
DRTT_L	Dentatorubrothalamic Tract Left	0.08
EMC_L	Extreme Capsule Left	0.79
EMC_R	Extreme Capsule Right	0.99
FAT_L	Frontal Aslant Tract Left	0.04
ICP_R	Inferior Cerebellar Peduncle Right	0.15
IFOF_L	Inferior Fronto-occipital Fasciculus Left	0.43
ILF_L	Inferior Longitudinal Fasciculus Left	0.10
ML_L	Medial Lemniscus Left	0.02
MdLF_L	Middle Longitudinal Fasciculus Left	0.34
MdLF_R	Middle Longitudinal Fasciculus Right	0.94
PAT_L	Parietoangular Tract Left	0.05
SLF2_L	Superior Longitudinal Fasciculus II Left	0.16
SLF2_R	Superior Longitudinal Fasciculus II Right	0.34
SLF3_L	Superior Longitudinal Fasciculus III Left	0.08
SLF3_R	Superior Longitudinal Fasciculus III Right	0.09
TR_S_R	Thalamic Radiation Superior Right	0.10
UF_L	Uncinate Fasciculus Left	0.21
V	Vermis	0.42
VOF_L	Vertical Occipital Fasciculus Left	0.43

Supplementary Table 11: Disrupted white matter tracts of the somatosensory WM mask.

Abbreviation	Full Name	Magnitude of disruption
AC	Anterior Commissure	0.23
AF_R	Arcuate Fasciculus Right	0.79
CC	Corpus Callosum	0.12
CPT_P_R	Corticopontine Tract Parietal Right	0.53
CST_R	Corticospinal Tract Right	0.33
EMC_L	Extreme Capsule Left	0.16
EMC_R	Extreme Capsule Right	0.90
IFOF_L	Inferior Fronto-occipital Fasciculus Left	0.04
IFOF_R	Inferior Fronto-occipital Fasciculus Right	0.14
ILF_L	Inferior Longitudinal Fasciculus Left	0.04
ILF_R	Inferior Longitudinal Fasciculus Right	0.16
ML_R	Medial Lemniscus Right	0.39
MdLF_R	Middle Longitudinal Fasciculus Right	0.37
PAT_R	Parietoangular Tract Right	0.03
SLF2_R	Superior Longitudinal Fasciculus II Right	0.25
SLF3_R	Superior Longitudinal Fasciculus III Right	0.42
TR_S_R	Thalamic Radiation Superior Right	0.09
VOF_L	Vertical Occipital Fasciculus Left	0.20

Supplementary Table 12: Disrupted white matter tracts of the attention WM mask.

Abbreviation	Full Name	Magnitude of disruption
AC	Anterior Commissure	0.03
AF_L	Arcuate Fasciculus Left	0.01
AF_R	Arcuate Fasciculus Right	0.75
AR_R	Acoustic Radiation Right	0.18
CBT_L	Corticobulbar Tract Left	0.65
CBT_R	Corticobulbar Tract Right	0.76
CC	Corpus Callosum	0.11
CPT_F_L	Corticopontine Tract Frontal Left	0.27
CPT_F_R	Corticopontine Tract Frontal Right	0.93
CPT_P_R	Corticopontine Tract Parietal Right	0.36
CST_L	Corticospinal Tract Left	0.40
CST_R	Corticospinal Tract Right	0.54
CS_A_R	Corona Radiata Anterior Right	0.16
CS_S_L	Corona Radiata Superior Left	0.23
CS_S_R	Corona Radiata Superior Right	0.44
C_FP_L	Cingulum Frontal Parietal Left	0.19
C_FP_R	Cingulum Frontal Parietal Right	0.08
DRTT_L	Dentatorubrothalamic Tract Left	0.04
DRTT_R	Dentatorubrothalamic Tract Right	0.27
EMC_L	Extreme Capsule Left	0.18
EMC_R	Extreme Capsule Right	0.96
FAT_L	Frontal Aslant Tract Left	0.12
FAT_R	Frontal Aslant Tract Right	0.58
IFOF_L	Inferior Fronto-occipital Fasciculus Left	0.01
IFOF_R	Inferior Fronto-occipital Fasciculus Right	0.35
ILF_L	Inferior Longitudinal Fasciculus Left	0.03
ILF_R	Inferior Longitudinal Fasciculus Right	0.08
ML_L	Medial Lemniscus Left	0.06
ML_R	Medial Lemniscus Right	0.18
MdLF_R	Middle Longitudinal Fasciculus Right	0.85
PAT_R	Parietoangular Tract Right	0.58
RST_R	Rubrospinal Tract Right	0.29
SLF1_L	Superior Longitudinal Fasciculus I Left	0.69
SLF1_R	Superior Longitudinal Fasciculus I Right	0.05
SLF2_R	Superior Longitudinal Fasciculus II Right	0.20
SLF3_R	Superior Longitudinal Fasciculus III Right	0.24
TR_S_R	Thalamic Radiation Superior Right	0.25
UF_R	Uncinate Fasciculus Right	0.16

Supplementary Table 13. Validation of CPMs against RAPID T-max maps: patient-level voxel-wise Spearman correlation coefficients (ρ) across 99 patients. Note that * denotes significant (p-value < 0.05)

Subject No#	Spearman rho
S01	0.72*
S02	0.79*
S03	0.83*
S04	0.79*
S05	0.89*
S06	0.90*
S07	0.82*
S08	0.79*
S09	0.82*
S10	0.68*
S11	0.81*
S12	0.77*
S13	0.82*
S14	0.87*
S15	0.78*
S16	0.83*
S17	0.83*
S18	0.81*
S19	0.69*
S20	0.82*
S21	0.91*
S22	0.67*
S23	0.94*
S24	0.85*
S25	0.83*
S26	0.83*
S27	0.79*
S28	0.84*
S29	0.81*
S30	0.89*
S31	0.87*
S32	0.86*
S33	0.80*
S34	0.91*
S35	0.77*
S36	0.88*
S37	0.77*
S38	0.84*
S39	0.85*
S40	0.91*
S41	0.80*
S42	0.79*
S43	0.82*
S44	0.86*
S45	0.79*
S46	0.92*
S47	0.81*
S48	0.87*
S49	0.79*
S50	0.84*
S51	0.88*
S52	0.72*

S53	0.88*
S54	0.79*
S55	0.89*
S56	0.79*
S57	0.79*
S58	0.81*
S59	0.90*
S60	0.77*
S61	0.83*
S62	0.86*
S63	0.86*
S64	0.77*
S65	0.77*
S66	0.82*
S67	0.88*
S68	0.84*
S69	0.78*
S70	0.76*
S71	0.92*
S72	0.84*
S73	0.68*
S74	0.86*
S75	0.90*
S76	0.62*
S77	0.79*
S78	0.83*
S79	0.89*
S80	0.82*
S81	0.75*
S82	0.73*
S83	0.74*
S84	0.88*
S85	0.87*
S86	0.77*
S87	0.82*
S88	0.86*
S89	0.73*
S90	0.79*
S91	0.82*
S92	0.85*
S93	0.76*
S94	0.92*
S95	0.80*
S96	0.78*
S97	0.76*
S98	0.87*
S99	0.85*
**Average**<br>**SD**	**0.82**<br>**0.06**

Supplementary Table 14: Percentage of scores for each NIHSS sub-category.

NIHSS Sub-category	Score					Shannon Entropy
	0	1	2	3	4	
LOC-Arrival	73.51	20.60	4.81	1.08		1.08
LOC-Question	46.59	15.43	37.98			1.46
LOC-Command	70.50	13.14	16.37			1.17
Gaze	61.95	22.61	15.43			1.33
Visual	55.06	22.54	20.10	2.30		1.55
Facial Palsy	25.70	30.80	38.91	4.59		1.76
Left motor hand	50.47	14.86	7.97	8.26	18.45	1.94
Right motor hand	57.29	15.22	5.74	7.54	14.21	1.79
Left motor leg	46.66	16.30	10.55	11.77	14.72	2.05
Right motor leg	53.19	14.14	9.33	10.19	13.14	1.92
Ataxia	76.53	14.83	8.61			1.01
Somatosensory	51.11	30.22	18.66			1.47
Language	45.73	18.74	18.16	17.37		1.85
Dysarthria	28.93	45.66	25.41			1.54
Attention	53.33	32.52	14.14			1.41

Supplementary Table 15. Sensitivity analysis of WM tract disruption thresholds. This table presents the estimated 2D cross-correlation coefficient and SSIM across eight conditions, each defined by a different threshold used to determine whether a WM tract was disrupted. In the first row, we estimated the 2D cross-correlation coefficient and SSIM between the 2D matrix for condition 1 and the average 2D matrix. In condition 1, a WM tract was considered disrupted if at least two WM voxels were located within 2 mm of the tract (see Supplementary Fig. 14). The average 2D matrix was generated with a similar layout to Supplementary Fig. 14 but averaged across all eight conditions.

Condition	Number of disruption	2D-cross correlation coefficient	Structural similarity index measure (SSIM)
1	2	0.9750	0.8912
2	4	0.9860	0.9487
3	6	0.9940	0.9725
4	8	0.9974	0.9839
5	10	0.9955	0.9750
6	12	0.9927	0.9601
7	14	0.9854	0.9263
8	16	0.9789	0.8979
	**Average**	**0.9881**	**0.9445**
	**SD**	**0.0082**	**0.0356**
